# Evidence of Presynaptic Localization and Function of the c-Jun N-Terminal Kinase

**DOI:** 10.1155/2017/6468356

**Published:** 2017-03-07

**Authors:** Silvia Biggi, Lucia Buccarello, Alessandra Sclip, Pellegrino Lippiello, Noemi Tonna, Cristiano Rumio, Daniele Di Marino, Maria Concetta Miniaci, Tiziana Borsello

**Affiliations:** ^1^IRCCS Istituto di Ricerche Farmacologiche “Mario Negri”, Via La Masa 19, 20156 Milano, Italy; ^2^Department of Pharmacy, University of Naples Federico II, Naples, Italy; ^3^Sanipedia S.r.l., Via Ariosto 21, 20091 Bresso, Italy; ^4^Department of Pharmacological and Biomolecular Sciences, University of Milan, Milano, Italy; ^5^Department of Informatics, Institute of Computational Science, Università della Svizzera Italiana (USI), Via G. Bu 13, 6900 Lugano, Switzerland

## Abstract

The c-Jun N-terminal kinase (JNK) is part of a stress signalling pathway strongly activated by NMDA-stimulation and involved in synaptic plasticity. Many studies have been focused on the post-synaptic mechanism of JNK action, and less is known about JNK presynaptic localization and its physiological role at this site. Here we examined whether JNK is present at the presynaptic site and its activity after presynaptic NMDA receptors stimulation. By using N-SIM Structured Super Resolution Microscopy as well as biochemical approaches, we demonstrated that presynaptic fractions contained significant amount of JNK protein and its activated form. By means of modelling design, we found that JNK, via the JBD domain, acts as a physiological effector on T-SNARE proteins; then using biochemical approaches we demonstrated the interaction between Syntaxin-1-JNK, Syntaxin-2-JNK, and Snap25-JNK. In addition, taking advance of the specific JNK inhibitor peptide, D-JNKI1, we defined JNK action on the SNARE complex formation. Finally, electrophysiological recordings confirmed the role of JNK in the presynaptic modulation of vesicle release. These data suggest that JNK-dependent phosphorylation of T-SNARE proteins may have an important functional role in synaptic plasticity.

## 1. Introduction

The c-Jun NH_2_-terminal protein kinase (JNKs) is part of the mitogen-activated protein kinase (MAPK) family [1, 2] and is represented in mammals by three genes coding for three isoforms: JNK1, JNK2, and JNK3. JNK1 and JNK2 expression is essentially ubiquitous, while JNK3 presence is restricted to brain and testis [3]. JNKs activation is led by several types of extracellular stress stimuli such as heat shock, UV irradiation, inflammatory cytokines, A*β* oligomers-induced toxicity, and excitotoxicity, primarily leading to cell death.

JNK phosphorylates numerous important substrates, including transcription factors AP-1, c-Jun, and Fos, leading to activation of nuclear response [3–5]. Furthermore, JNK regulates intracellular signalling pathways phosphorylating many cytosolic and cell-membrane targets. Many data pointed out that the JNK activation in the brain is higher than in any other mammalian tissue [6–8], suggesting that kinase might play an important role in the central nervous system (CNS), regulating both physiological and pathological brain processes.

JNK activation, in fact, induces neuronal death in a wide range of pathological conditions, such as NMDA-induced excitotoxicity, ischemic stroke [9–11], and traumatic brain injury [12]; JNK activation has been also associated to many neurodegenerative disorders like Alzheimer [13–18], Huntington's [19], and Parkinson's disease [20, 21], but also psychiatric disorders and intellectual disabilities involving synaptic structure anomalies [22]. On the other hand, JNK plays also an important role in regulating physiological processes such as neuronal precursors migration, axonal sprouting and polarization, dendritic branches elongation, dendritic spines stabilization, and therefore neuronal plasticity and regeneration in the adult brain.

All these evidences point out that JNK pathway in the CNS is particularly intricate, since it is not only subjected to different types of stimulations, which modulate JNK response, but it also plays different roles depending on its localization, having JNK pool distinct functions in the various cellular districts. Since neurons are exquisitely polarized cells, it is plausible that JNK activation may have different roles and functions in diverse subcellular localizations, from nuclear to cytoplasmic compartments as well as axons, dendrites, and dendritic spines. This effect is not only due to compartmentalization, but also due to the huge variety of JNK's targets. For instance c-Jun, the JNK elective target, is localized in the nucleus and regulates gene expression, while microtubule associated proteins (MAPs) are in the cytoplasm [23–26] and control axon elongation and dendrite branching [22]. More recently, JNK has been also identified at synaptic sites [27–29]; however its role in this district as well as its activation level is not completely clear yet.

Different works suggested that JNK is preferentially located in postsynaptic regions, where it interacts with key synaptic scaffold proteins (PSD-95 and PSD-93) as well as with GluR2 AMPAR subunit (GluR2L) [30]. We demonstrated that JNK has an important role in regulating glutamate receptors expression at postsynaptic density level after A*β*-induced toxicity in hippocampal neurons [17, 31]. Furthermore, many lines of evidence demonstrated the importance of NMDA receptors (NMDARs) as JNK activators in the postsynaptic compartment [9, 32], suggesting that JNK might have a crucial role in synaptic plasticity as well as in excitotoxicity. NMDA-induced excitotoxicity in fact, mediated mainly by postsynaptic NMDARs, powerfully activates JNK, and JNK specific inhibition prevents NMDAR-mediated neuronal death in cortical neurons [9, 10, 25] as well as in organotypic hippocampal slices culture [9]. However, less is known about the biological role of presynaptic NMDARs (pre-NMDARs) and their downstream pathways. The pre-NMDARs have been extensively characterized in the last years as new key-players during development and memory consolidation phases. They are mainly involved in synaptic strength modulation, through mechanisms ranging from spontaneous neurotransmitter release facilitation to LTP/LTD [33–39]. The pre-NMDARs are abundantly expressed in the developing nervous system [40–42] although their expression decreases in the adult, persisting only in most plastic brain areas (such as neocortex, cerebellum, and hippocampus) [43]. Importantly, their aberrant activation has been also associated to pathological events [44, 45]. Although JNKs presence in postsynaptic compartment has been characterized, there is lack of data for their presence and physiological role at presynaptic sites. Here we investigate JNK localization and role in presynaptic compartment. JNK identification at the presynaptic site and its activation after NMDARs stimulation may be important not only to clarify this issue, but also for the identification of new targets contributing to synaptic plasticity. Moreover, JNK downstream pathway definition could represent a new therapeutic tool to prevent synaptic dysfunction in neurodegenerative diseases.

## 2. Materials and Methods 

### 2.1. Purified Synaptosomes Extraction

Purified synaptosomes from mice cortex were extracted as in Dunkley [38]. Briefly, tissues were gently homogenated with a glass-Teflon grinder in ST buffer (sucrose 320 mM, Tris 10 mM; pH 7.4) supplemented with protease and phosphatase inhibitors. Homogenates were centrifuged (1.000*g* 5 min 4°C) and supernatants were collected and centrifuged again (13.000 rpm, 20 min, 4°C). Pellet was resuspended in ST buffer and stratified on a 20–10–6–2% Percoll (GE Healthcare) gradient in ST buffer, prepared in ultracentrifuge tubes. Samples were then ultracentrifuged, for 8 min 33500*g* 4°C and interface between 10% and 20% layers, containing purified synaptosomes, was aspired. Purified synaptosomes were then precipitated 20 min 10.000*g* 4°C in 6 : 1 vol/vol Hepes Buffer (NaCl 140 mM, KCl 3 mM, MgSO_4_ 1.2 mM, CaCl_2_ 1.2 mM, NaH_2_PO_4_ 1 mM, NaHCO_3_ 3.5 mM, glucose 10 mM, Hepes 5 mM; pH 7.4, Sigma). Pellet was lysed or resuspended in the appropriate buffer.

### 2.2. Crude Synaptosomes Extraction

Crude synaptosomes were used for calcium currents evaluation. Brains were homogenated with a glass-Teflon grinder in SHE buffer (Hepes 4 mM pH 7.3, sucrose 320 mM, EGTA 1 mM, Sigma) with protease inhibitors and phosphatase inhibitors. After a first centrifuge (10.000*g* 10 min 4°C), supernatant was collected and again centrifuged (12.500*g*, 20 min 4°C). Pellet was resuspended in SHE buffer and subjected to a third centrifuge (12.500*g*, 20 min, 4°C) in order to obtain crude synaptosomal fraction pellet, which was resuspended in KRH buffer (NaCl 125 mM, KCl 5 mM, MgSO_4_ 1.2 mM, KH_2_PO_4_ 1.2 mM, Hepes NaOH pH 7.4 25 mM, glucose 6 mM, CaCl_2_ 2 mM, Sigma) and kept at 37°C 5% CO_2_ for 45 min, for functional reactivation. Crude reactivated synaptosomes were then resuspended at a final concentration of 1 mg–500 *μ*g/ml in KRH. After resuspension, synaptosomes were kept at 37°C 5% CO_2_ for 25 min for an habituation step.

### 2.3. Calcium Currents Evaluation

Calcium currents were measured on crude synaptosomes. Briefly, after reactivation step, synaptosomes were pelleted as described before, resuspended in 300 *μ*l 1 : 1 mixture using KRH and calcium sensitive cell-permeating fluorophore Fluo-4 (Fluo-4 Direct™ Calcium Assay, Life Technologies). Samples were placed in a whole black 96-well (Corning Costar® 96-Well Black Polystyrene Plate) for 25 min 37°C 5% CO_2_ to allow Fluo-4 to permeate cell membranes. After habituation fluorescence was measured with a Tecan F500 spectrophotometer for 180 cycles (corresponding to approximately 7 minutes) at 37°C. NMDA (100 *μ*M, with glycine coagonist 1 *μ*M final concentration) or KCl (50 mM final concentration) were injected 100 *μ*l/sec at cycle 20 (45 sec). Fluorescence was recorded by a top mode reading optimizing* Z*-position.

### 2.4. Synaptosomes Stimulation

25 minutes before stimulation D-JNKI1 peptide (Xigen) is added to the medium at a final concentration of 2 *μ*M. Synaptosomes were treated with NMDA 100 *μ*M (Abcam) with glycine coagonist 1 *μ*M (Sigma) or KCl 50 mM (Sigma) for 2′, 5′, 7′, or 10′ at 37°C 5% CO_2_. For NMDA stimulation MgSO_4_ was omitted from the buffer. At the end of the stimulation synaptosomes were resuspended in lysis buffer. For western blotting analysis Bonny lysis buffer was used [25] while for SNARE complex evaluation SNARE buffer was used [37].

### 2.5. Immunoprecipitation

Immunoprecipitation was conducted on purified cortical synaptosomes as previously described [39]. Immunoprecipitation antibodies, all used at 1 : 100, were anti-JNK (#06-748, Upstate), anti-Syntaxin-1 (#A1238, Assay Biotech), anti-Syntaxin-2 (#110 022, Synaptic Systems), and anti-Snap25 (#331, Chemicon). After precipitation process 0.5–1 *μ*g proteins were subjected to SDS-Page.

### 2.6. SDS-PAGE

20 *μ*g of brain homogenates, TIF (Triton-Insoluble Fraction), extracted as in [40] crude/purified synaptosomal proteins, was separated by SDS (Bio-Rad) polyacrylamide gel electrophoresis (ProtoGel, National Diagnostics). For SNARE complex detection, samples (referred to as unboiled) were kept at RT for 7 min before loading. Antibodies used were anti-p-JNK and-JNK (#9251 and #9252 both Cell Signalling, 1 : 1000), anti-Syntaxin-1 (#78539, Abcam), anti-Syntaxin-2 (#S0664, Sigma, 1 : 5000), Snap25 (Stressgen, 1 : 5000), anti-Syntaxin-1 (Abcam, 1 : 5000), anti-Vamp (#104 211, Synaptic Systems), anti-Synaptophysin (#S5768, Sigma), anti-PSD95 (#10011435, Cayman Chemicals, 1 : 1000), and anti-Actin (1501, Millipore, 1 : 5000). Blots were developed using ECL chemiluminescence system (ECL Western Blotting Substrate, Promega) and quantified by densitometry using ImageJ software.

### 2.7. Immunofluorescence

After extraction, purified synaptosomes were processed as in Sokolow [41]. Primary antibodies used were anti-Synaptophysin 1 : 500 (#S5768, Sigma) and anti-p-JNK 1 : 100 (#6254, Santa Cruz) while fluoresceinated secondary antibodies were Alexa Fluor 488 anti-Mouse and Alexa Fluor 594 anti-Rabbit antibodies. Images were acquired with Super-Resolution microscope (N-SIM, Nikon-Structured Illumination Microscopy). Protein colocalization was analysed using Pearson's colocalization coefficient with Imaris software.

### 2.8. Acute Slice Preparation

Experiments were performed on horizontal slices prepared from the somatosensory cortex of CD-1 mice of either sex, 2-3-month-old. Each animal was anaesthetized with isoflurane, USP (Abbott Laboratories, Illinois, USA), and decapitated. The whole brain was removed and rapidly immersed in ice-cold extracellular saline solution containing (mM): Tris-Hcl 72, Tris-Base 18, NaH_2_PO_4_ 1.2, NaHCO_3_ 30, KCl 2.5, Glucose 25, HEPES 20, MgSO_4_ 10, Na-PIR 3, ascorbic acid 5, CaCl_2_ 0.5, sucrose 20, pH 7.4. Horizontal somatosensory slices (200 *µ*m thickness) were obtained using a vibratome (Vibroslice 752, Campden Instruments, Loughborough, UK). The slices were placed in a solution containing (mM): 125 NaCl, 2.5 KCl, 2 CaCl_2_, 1 MgCl_2_, 1.25 NaH_2_PO_4_, 26 NaHCO, 20 glucose, and the pH was maintained at 7.4 for 25/30 min a 32°C and then maintained at room temperature.

### 2.9. Electrophysiology

After a recovery period of at least 1 h, an individual slice was transferred to a recording chamber and continuously perfused with extracellular solution at room temperature (22/25°C). Whole-cell voltage-clamp recordings were made from cortical neurons using an EPC-8 patch-clamp amplifier (HEKA Elektronik, Lambrecht/Pfalz, Germany). Pipettes of borosilicate glass, with a tip resistance between 2.0 and 3 MΩ, were used for patch-clamp recordings [42]. The intracellular solution had the following composition (mM): K-gluconate 117, KCL 13, MgCl_2_·6H_2_0 2, Hepes 10, CaCl_2_ 1, EGTA 11, Na_2_ATP 2, Na_3_GTP 0.5, pH 7.3. Voltage-clamp recordings were accepted only if the series resistance was less than 10 MΩ. Data were filtered at 3 kHz and digitized at 10 kHz using the filter and analog/digital converter of the amplifier. Digitized data were stored on computer disk using the Pulse software (HEKA Elektronik).

### 2.10. Statistical Analysis

At least three independent replicates have been performed for each experiment. Statistical analysis was done using Graph Pad Prism 6 program. Data were analysed using Student's* t*-test and one- or two-way ANOVA, followed by Dunnet/Tukey's post hoc test. All data were shown as mean ± SEM with statistical significance given at *p* < 0.05.

### 2.11. Computational Analysis

The pairwise alignment between Syntaxin-1 (Uniprot ID Q16623) and Syntaxin-2 (Uniprot ID P32856) was performed using Needle software available in the European Molecular Biology Open Software Suit (EMBOSS) (PMID: 10827456).

The structure of Syntaxin-1 for the docking simulations with JNK1 was extracted from the Syntaxin-1-Munc18A complex (10.1038/emboj.2008.37). The structure of JNK1 complexed with a small peptide belonging to JIP scaffold protein [43] was selected for the docking simulations. The structure of Snap25 was extracted from the 20S supercomplex [44] while the same JNK1 structure selected for the docking with Syntaxin-1 was also used. The docking simulation was performed using HADDOCK 2.2 [45] program that is one of the most suitable software to correctly predict the protein-protein complexes. The images have been obtained using the software UCSF Chimera v.1.10.1.

## 3. Results

### 3.1. JNK Is Located in the Presynaptic Compartment

**Figure 1:**
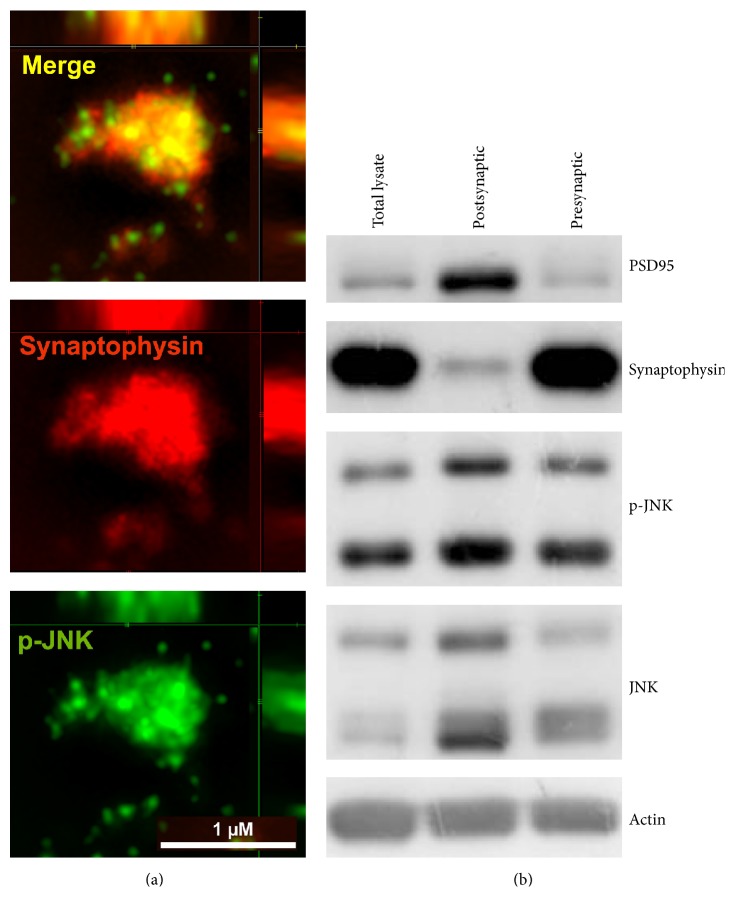
JNK is localized in the presynaptic compartment. (a) Immunofluorescence on mouse cortical purified synaptosomes stained for p-JNK (green) and for Syntaxin-2 (red) colocalization is visualized as yellow signal in Merge image, with Pearson's coefficient 0.54 ± 0.08. Images acquired with super resolution microscopy; scale bar 1 *μ*m. (b) Western blotting analysis of JNK and p-JNK active form presence in 20 *μ*g of total cortical lysates, postsynaptic density region (represented by PSD95 postsynaptic specific protein on TIF fraction) and presynaptic compartment (represented by Synaptophysin presynaptic specific protein on purified synaptosomes). JNK is localized both in postsynaptic and in presynaptic region and its phosphorylation state is comparable. Actin was used as loading control.

To investigate JNK localization in presynaptic compartment, we used N-SIM Structured Super Resolution Microscopy on isolated cortical synaptosomes (Figure 1(a)). Synaptosomes were double stained for Synaptophysin, a constitutive and specific presynaptic protein (labeled in red) and for the JNK active form p-JNK (labeled in green, Figure 1(a)). As shown in Figure 1(a) (Merge panel), the JNK active form p-JNK was located in presynaptic compartment and colocalized with Synaptophysin. This evidence is also confirmed by Pearson's coefficient value, corresponding to 0.54 ± 0.08.

JNK presynaptic localization was further investigated comparing the presynaptic to the postsynaptic fraction and to total brain homogenate. JNK and activated form p-JNK were evaluated by western blotting on total cortical homogenate, cortical synaptosomal lysate (presynaptic compartment), and TIF fractionation (postsynaptic compartment; Figure 1(b)). The P-JNK activated form, as well as the JNK total form, was detectable in the presynaptic compartment (Figure 1(b)) confirming the Super Resolution Microscopy data (Figure 1(a)). In the postsynaptic TIF fractionation, identified by PSD95 scaffold protein, JNK was present as expected. The purity of the synaptosomes was confirmed by the low presence of PSD-95 in the presynaptic fraction, which as described by Evans [46] is accountable to the nonfunctional PSD structure bound to active zone. Furthermore TIF fraction purity was proved by the low presence of Synaptophysin in postsynaptic fractions.

### 3.2. JNK Is Rapidly Activated after Pre-NMDARs Stimulation Both in Young and in Adult Mice

To investigate pre-NMDAR-mediated JNK activation, we evaluated p-JNK/JNK ratio by western blotting on cortical synaptosomes purified from young (p14) and adult mice after 2′, 5′, and 10′ minutes of treatment with NMDA (100 *μ*M) and glycine (1 *μ*M).

**Figure 2:**
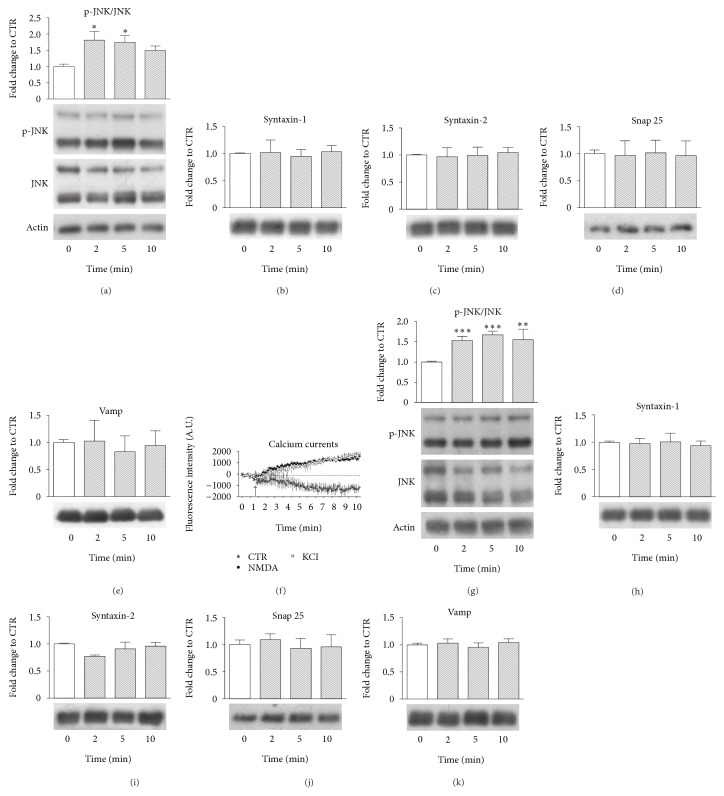
JNK is activated after pre-NMDARs stimulation in young mice and adult. (a–e) Western blotting and relative quantification of JNK activation (a), measured as p-JNK/JNK ratio, on p14 mice crude synaptosomes stimulated with NMDA 100 *μ*M and glycine 1 *μ*M for 2, 5, and 10 min. JNK activation significantly increases 2 min after treatment (^*∗*^*p* < 0.05) and persists at 5 min (^*∗*^*p* < 0.05). Western blotting and relative quantification of presynaptic proteins Syntaxin-1 (b), Syntaxin-2 (c), Snap25 (d), and Vamp (e) extracted from p14 mice crude synaptosomes stimulated with NMDA 100 *μ*M and glycine 1 *μ*M for 2, 5, and 10 min. Protein levels remain unchanged after treatment at the considered time-points. Actin was used as loading control (20 *μ*g proteins loaded; *N* = 5). One-way ANOVA,* Dunnet's* post-hoc test. Data are showed as mean ± S.E.M. (f) Calcium currents, measured with calcium sensible fluorophore Fluo4, on crude synaptosomes treated with NMDA 100 *μ*M and glycine 1 *μ*M or with KCl 50 mM. Fluorescence was recorded with a spectrophotometric approach for 7 min, with NMDA + Gly or KCl injection at 45 sec. After both injections there was a significant increase in calcium levels starting from 45 sec after stimulation (each experimental group *N* = 5). One-way ANOVA,* Dunnet's *post hoc test. Data are shown as mean ± S.E.M. (g–k) Western blotting and relative quantification of JNK activation (g), measured as p-JNK/JNK ratio on adult mice crude synaptosomes stimulated with NMDA 100 *μ*M and glycine 1 *μ*M for 2, 5, and 10 min. JNK activation increases 2 min after treatment (^*∗∗∗*^*p* < 0.001) and persists at 5 min (^*∗∗∗*^*p* < 0.01) and at 10 min (^*∗∗*^*p* < 0.01). Western blotting and relative quantification of presynaptic proteins Syntaxin-1 (h), Syntaxin-2 (i), Snap25 (j), and Vamp (k) extracted from adult mice crude synaptosomes stimulated with NMDA 100 *μ*M and glycine 1 *μ*M for 2, 5, and 10 min. Protein levels remained unchanged after treatment for all three time-points. Actin was used as loading control (20 *μ*g proteins loaded; *N* = 4). One-way ANOVA,* Dunnet's* post hoc test. Data are shown as mean ± S.E.M.

In young mice synaptosomes (Figure 2(a)), JNK was significantly activated after 2 minutes of NMDA stimulation (75% activation increase, ^*∗*^*p* < 0.05) and persisted after 5 minutes (70% activation increase, ^*∗*^*p* < 0.05; Figure 2(a)). No significant alteration in the levels of presynaptic protein was detected by western blotting (Figures 2(b)–2(e)), suggesting that, at the considered time-points (2′, 5′, and 10′ minutes), JNK activation did not determine the activation of protein degradation processes.

As previously described, pre-NMDARs expression decreases during neurodevelopment [43]: to verify if pre-NMDAR-mediated JNK activation was conserved in adult mice, synaptosomes were extracted from aged mice. To test if residual pre-NMDARs expression was enough to trigger depolarization, calcium currents were measured on synaptosomes by pre-ncubation with Fluo4 cell-permeant calcium sensitive dye. Synaptosomes were stimulated with NMDA (100 *μ*M) and glycine (1 *μ*M) or KCl 50 mM as positive control (Figure 2(f)). Recording of calcium influx was performed with a spectrophotometer for 7 minutes, corresponding to 180 fluorescence-reading cycles, with stimulus injection at 45 sec (cycle 20, Figure 2(f) black arrow). This time-point has been chosen as a compromise to avoid aberrant fluorescence measurement due to homeostasis loss caused by lack of CO_2_ and humidity control in the spectrophotometer chamber. As expected KCl injection led to a rapid and significant increase of intrasynaptosomal calcium levels lasting over time. Similarly, NMDA administration led to a comparable significant increase in fluorescence if compared to controls injected with vehicle, starting 45 sec (20 cycles) after injection and remaining stable over time. Unexpectedly we observed a drop in fluorescence level in vehicle injected synaptosomes (CTR); however, in line with previous data, NMDA+ Gly stimulation led to rapid JNK activation at 2 min (50% increase ^*∗∗∗*^*p* < 0.001), which increases at 5 min (60% increase, ^*∗∗∗*^*p* < 0.001) and persisted at 10 min (50% increase, ^*∗∗*^*p* < 0.01; Figure 2(g)). These data suggested that pre-NMDAR-JNK axis was conserved during adult life. In fact, pre-NMDARs retained the ability to trigger excitatory response. The stimulation with NMDA (100 *μ*M) and glycine (1 *μ*M) induced JNK activation in both young (p14) and adult synaptosomes, and the consequent p-JNK/JNK ratio was comparable. As for young synaptosomes, also in this case, no significant protein degradation events were observed: no significant change of presynaptic proteins levels (Syntaxin-1 and Syntaxin-2, Snap25, and Vamp) at the considered time-points (2′ 5′ and 10′) was found (Figures 2(h)–2(k)).

### 3.3. Investigation of JNK Possible Targets

**Figure 3:**
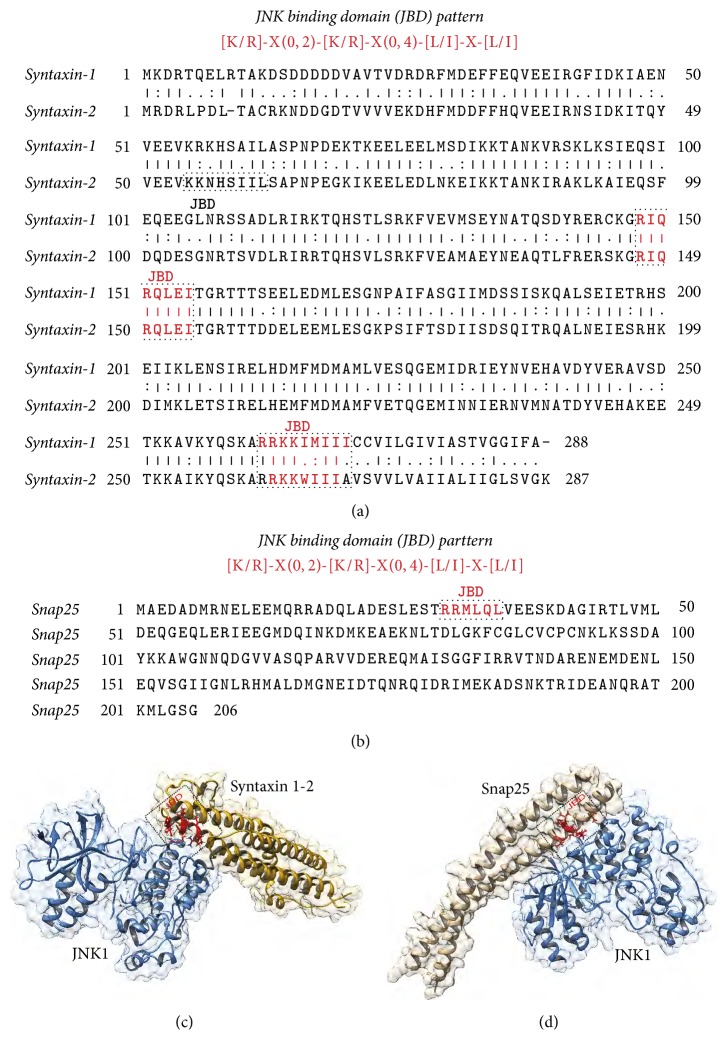
JBD is crucial for SNARE interaction with JNK. (a) Sequence alignment between Syntaxin-1 and Syntaxin-2. The JBDs are underlined in red. (b) Sequence of Snap25 with the JBD underlined in red. On top of panels (a) and (b) the canonical JBD pattern is also reported. (c) Best JNK-Syntaxin complex resulting from the docking predictions. JNK is shown in light blue and Syntaxin in gold. The lateral chains of the residues belonging to the JBD are shown in red. (d) Best complex of the JNK-Snap25 docking results. Also in this case JNK is depicted in light blue while Snap25 is shown in light brown. The lateral chains of the residues of the JBD in Snap25 are reported in red.

To get more insight into JNK modulation of vesicle release, JNK binding domain (JBD) was searched among presynaptic machinery proteins amino acidic sequences. In recent years, the extensive characterization of the JBDs in several JNK substrates [5] allowed identification of a canonical amino acid pattern for the JNK-substrate interaction. This canonical pattern is composed of two positively charged and two hydrophobic residues separated by two spacer regions with different length: [KR]-X_ (0,2)_-[KR]-X_ (0,4)_-[LI]-X- [LI] [47]. Among the critical steps involved in neurotransmitter release we decided to consider vesicle docking and priming steps, since previously reported data showed that JNK specific inhibition is not associated to any modification in calcium influx after NMDAR stimulation [25]. Thus it is unlikely that JNK could act on modulating this process in the presynaptic compartment. As reported in Figure 3(a), there are two possible JBDs in Syntaxin-1 and three in Syntaxin-2, respectively. Two out of three possible JBDs are conserved between Syntaxin-1 and Syntaxin-2 (Figure 3(a)). In particular, the JBD among residues 148–155 is fully conserved between the two isoforms (Figure 3(a)) making this region a very good candidate to be involved in the interaction with JNK. Analyzing also the sequence of Snap25, a unique putative JBD has been found (Figure 3(b)). The presence of putative JBDs pattern in three presynaptic machinery proteins is a strong indication of possible physical interaction of those proteins with JNK.

### 3.4. JNK Interacts with SNARE Proteins

The presence of JBDs sequences in three members (Syntaxin-1, Syntaxin-2, and Snap25) of the SNARE protein family allowed us to predict the possible three-dimensional (3D) organization of the JNK-SNARE protein complexes using computational approach, the protein-protein docking (Figures 3(c)-3(d)). The JBD sequence, fully conserved in both isoforms of Syntaxin (residues 148–155), has been mapped on the structure of Syntaxin-1 [48], which we have chosen as a representative structure also for Syntaxin-2, because they share a sequence identity of 64.4% and sequence similarity of 80% (Figure 3(c)). The unique JBD sequence located between residues 30 and 35 in Snap25 was also mapped on the 3D structure (Figure 3(d)).

The JBD present on the SNARE proteins and the region of JNK1 contacting the JIP peptide was used to drive the docking simulation performed with HADDOCK 2.2 software [49]. The best Syntaxin-1-JNK and Snap25-JNK complexes were reported in Figures 3(c) and 3(d) with residues belonging to the JBDs underlined. Both complexes have a favourable HADDOCK score that is a sum of different energy contribution terms (i.e., electrostatic energy, Van der Waals energy, desolvation energy, and restraints violation energies). In detail, the Syntaxin-1-JNK best complex reports an HADDOCK score of −24.9 ± 6.2 while the Snap25-JNK1 reports an HADDOCK score of −115.3 ± 0.8, underlining that the second complex formation could be more favoured. In both complexes the JBDs are involved in a dense network of hydrophobic and electrostatic interaction with JNK that are crucial for the stability of the complexes.

**Figure 4:**
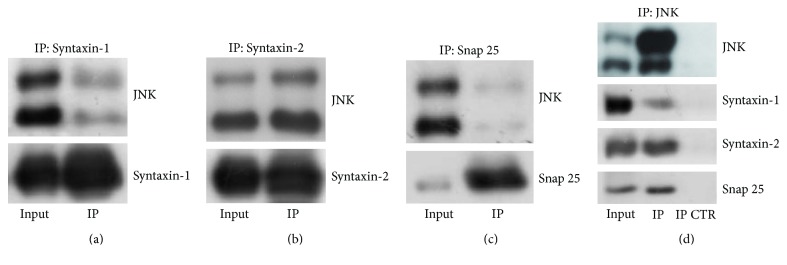
JNK preferentially interacts with Syntaxin-1, Syntaxin-2, and Snap25. (a–c) Western blotting after immunoprecipitation of Syntaxin-1 (a), Syntaxin-2 (b), and Snap25 (c) on purified synaptosomes lysates extracted from adult mice cortex. JNK is detectable in Syntaxin-1 and Syntaxin-2 precipitate, while immunoreactive signal is weak in Snap25 precipitate. (d) Both Syntaxin-1 and Syntaxin-2, as well as Snap25, were detectable in JNK precipitate. 500 *μ*g of total lysates has been subjected to immunoprecipitation, while 20 *μ*g was loaded as control. *N* = 4.

To prove these interactions, SNARE proteins were evaluated with western blotting, after immunoprecipitation of Syntaxin-1, Syntaxin-2, and Snap25 on purified cortical synaptosomal lysates. JNK was found in the coprecipitate; in particular the JNK protein band was more detectable with Syntaxin-2 and Syntaxin-1 and was less intensely visible with Snap25 (Figures 4(a), 4(b), and 4(c)). To confirm the interaction of JNK with Syntaxin-1, Syntaxin-2, and Snap25, the reverse immunoprecipitation on purified cortical synaptosomal lysates was performed. According to data previously found mostly Syntaxin-2 and Syntaxin-1 were traceable in JNK's coprecipitate (Figure 4(d)). However, a precise quantification is not possible due to the different efficiency of the antibodies used.

### 3.5. SNARE Proteins Interact Both with JNK2 and with JNK3

**Figure 5:**
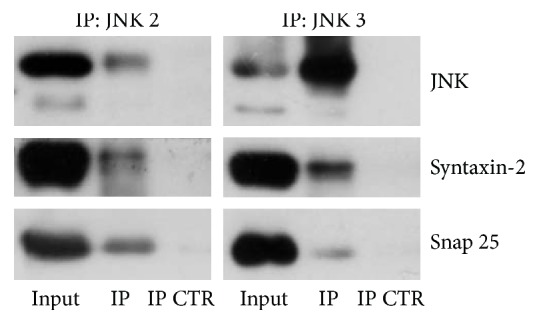
Both JNK2 and JNK3 interact with Syntaxin-2 and Snap25. Western blotting after immunoprecipitation of JNK2 (left) and JNK3 (right) on purified synaptosomes lysates extracted from adult mice cortex. Syntaxin-2 and Snap25 were detectable both in JNK2 and in JNK3 precipitate. 500 *μ*g of total lysates has been subjected to immunoprecipitation, while 20 *μ*g was loaded as control (each experimental group *N* = 3).

To investigate if a specific JNK isoform was involved in the interaction with SNARE protein complex, single JNK isoform precipitation was performed on cortical synaptosomal lysates. After precipitation of JNK3 [7, 50], an isoform known to be more sensitive to NMDA stimulation, and of JNK2, an isoform known to be important for neural plasticity, SNARE protein expression was evaluated by western blotting in the coprecipitate. Presence of both Syntaxin-2 and Snap25 was traceable in JNK2 coprecipitate (Figure 5, left panel); similarly both SNAREs were present in JNK3 coprecipitate (Figure 5, right panel). These results indicated that both JNK isoforms, JNK3 and JNK2, interact with Syntaxin and Snap25, without having a preferential interaction with a specific isoform.

### 3.6. JNK Inhibition Reduces the Frequency but Not the Amplitude of mEPSCs in Cortical Slices

**Figure 6:**
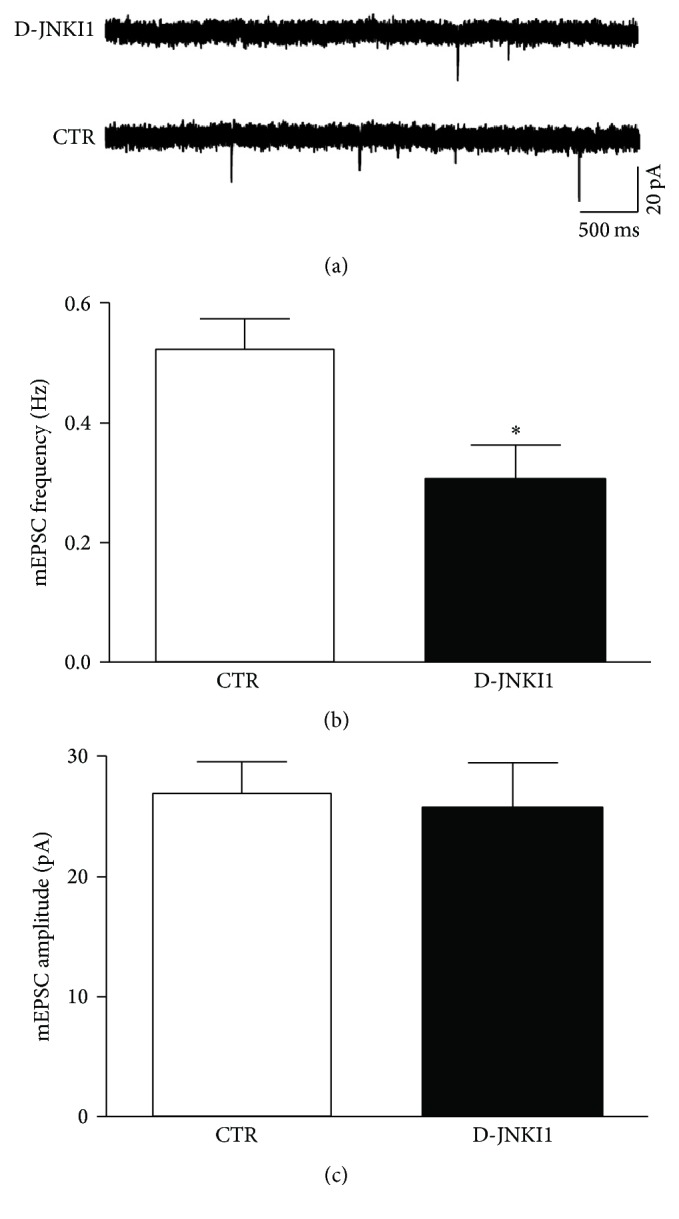
D-JNKI1 treatment reduced the frequency of mEPSCs in mice cortical slices. (a) Representative traces of mEPSCs recorded from cortical slices under control condition or after incubation with D-JNKI1n for 1 hr. (b-c) Bar graph (mean ± SEM) showing the mEPSC frequency (b) and amplitude (c) obtained in the first 5 min of recordings from control (*N* = 8) and D-JNKI1-treated (*N* = 8) cortical neurons. CTR versus D-JNKI1 treatment: ^*∗*^*p* < 0.05 Student's t-test, n =8. Data are shown as mean +/− SEM.

To determine the site of synaptic modifications induced by JNK, the miniature excitatory postsynaptic currents (mEPSCs) were examined in the presence of JNK inhibitor, D-JNKI1. Miniature synaptic events are due to the spontaneous release of glutamate vesicles, which in turn activates postsynaptic glutamate receptors. According to the quantal hypothesis, changes in the amplitudes of mEPSCs reflect a change in postsynaptic responsiveness, whereas presynaptic mechanisms result in no change in the amplitude of the mEPSCs but may cause a change in the frequency of mEPSCs recorded in the postsynaptic cell [51, 52]. The mEPSCs were, therefore, recorded in cortical slices, pretreated for 1 hr with D-JNKI1 (2 *µ*M), in the presence of TTX (1 *µ*M) to prevent action-potential firing, and bicuculline (20 *µ*M) to block GABAergic currents. As shown in Figure 6(a) (sample traces of mEPSCs) and in Figures 6(b)-6(c) (quantification of mEPSCs frequency and amplitude), D-JNKI1 induced a significant decrease in the frequency of mEPSCs if compared to control conditions (^*∗*^*p* < 0.05, *N* = 8) but had no statistically significant effect on mEPSCs amplitude. These results support the hypothesis that JNK can act presynaptically to regulate transmitter release.

### 3.7. JNK Inhibition Reduces SNARE Complex Formation after NMDA Stimulation

**Figure 7:**
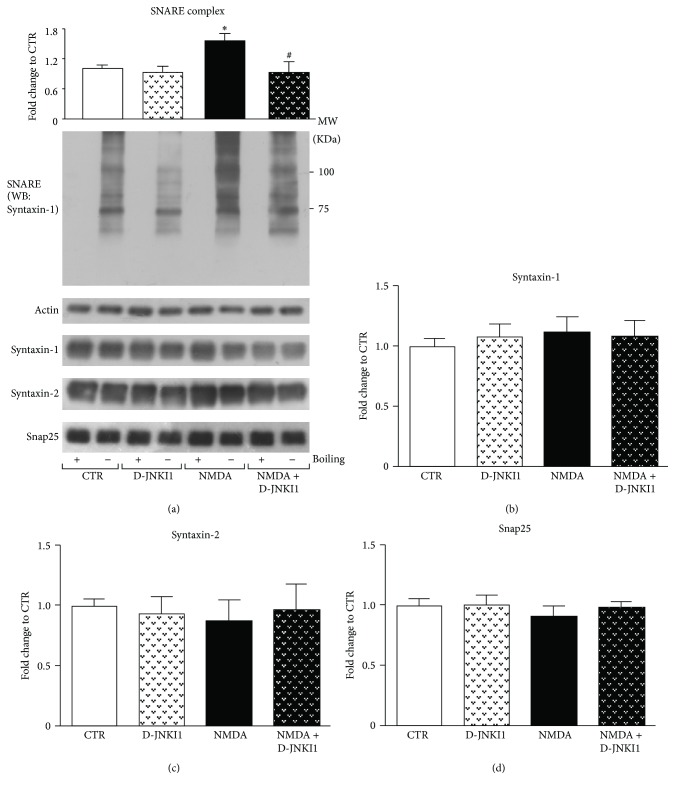
JNK inhibition reduces SNARE complex formation. (a) Western blotting and relative quantification of SNARE complex levels (anti-Syntaxin-1 immunoreactive bands from approximately 75 to 100 KDa) in 20 *μ*g of crude synaptosomes stimulated with NMDA 100 *μ*m and glycine 1 *μ*m for 7 min in presence or absence of D-JNKI1 (2 *μ*m), added to synaptosomes 25 min before stimulation. After NMDA/Gly exposure there was a significant increase in SNARE complexes formation (^*∗*^*p* < 0.05), which is totally prevented by D-JNKI1 preadministration (^#^*p* < 0.05). SNARE complexes are normalized on actin, *N* = 6. Data are shown as mean ± SEM, Two-way ANOVA,* Tukey's* post hoc test. (b–d) Western blotting and relative quantification of Syntaxin-1, Syntaxin-2, and Snap25 in 20 *μ*g of crude synaptosomes stimulated with NMDA 100 *μ*m and glycine 1 *μ*m for 7 min in presence or absence of D-JNKI1 (2 *μ*m), added to synaptosomes 25 min before stimulation. There was no significant variation of protein levels after NMDA/Gly exposure as well as after D-JNKI1 administration alone or in combination with NMDA/Gly. Proteins are normalized on actin, *N* = 6. Two-way ANOVA,* Tukey's* post hoc test. Data are shown as mean ± SEM.

Having demonstrated JNK interaction with Syntaxin-1/Syntaxin-2 as well as with Snap25 and that the specific inhibition of JNK, with D-JNKI1, reduced spontaneous release in an in vitro slice preparation. We here studied the effect of D-JNKI1 on JNK-SNARE complex formation in control condition and after NMDA stimulation. The SNARE complexes (Syntaxin + Snap25 + Vamp) are detergent resistant structures and can be visualized by western blotting on unboiled samples [53]. Anti-Syntaxin-1 antibody was used to visualize SNARE complex at approximately 75–100 KDa and used for densitometric quantification. Higher MW signals, ascribable to more structured complexes, enriched with other release-machinery factors, were detected too. The synaptosomes were treated with D-JNKI1 (2 *μ*M) for 25 minutes. Inhibition of JNK did not affect SNARE complex assembly in control condition. When synaptosomes were stimulated by NMDA (100 *μ*M NMDA plus 1 *μ*M glycine), there was a significant 50% increase of complex formation (^*∗*^*p* < 0.05) after 7 minutes of treatment, due to the increased vesicle release triggered by the pre-NMDARs activation (Figure 7(a)). When synaptosomes were firstly pretreated with D-JNKI1 (2 *μ*M) for 25 minutes and then stimulated with NMDA, SNARE complex assembly was significantly prevented (^#^*p* < 0.05) and complex levels were restored to nonstimulated control levels (see Figure 7(a)). These results showed that JNK inhibition prevented SNARE proteins complexation, according to electrophysiology data. Finally, the levels of SNARE complex proteins were evaluated in boiled samples for all the conditions, control synaptosomes with and without D-JNKI1 as well as synaptosomes stimulated by NMDA with and without D-JNKI1. No change in protein levels was observed under these conditions (Figures 7(b)–7(d)). However we were not able to detect any reduction of noncomplexed Syntaxin-1 in unboiled NMDA treated samples, even though a significant reduction could have been expected due to Syntaxin-1 assembly in SNARE complex. This might probably be due to the huge enrichment of Syntaxin-1 in synaptosomal fraction, whose alterations in the protein levels cannot be conclusively evaluated by western blotting.

## 4. Discussion

JNK kinase plays an important role in regulating several functions in the central nervous system and in synaptic plasticity processes [54, 55]. In particular, a specific JNK pool in the postsynaptic site is able to modulate synaptic strength regulating both AMPA and NMDA glutamate receptors localization in the postsynaptic density region (PSD). This feature is partially due to JNK interaction with PSD scaffold protein PSD95 [56], but also by its direct interaction with NR2A/B [57, 58]. Importantly, in pathological conditions, such as Alzheimer's disease (AD) synaptopathy, JNK induces a massive removal of glutamate receptors (AMPA and NMDA) from PSD region, leading to LTP impairment, LTD increase [18, 31], and dendritic spines loss [17, 31]. JNK's role in the regulation of glutamatergic synapse dysfunction and dysmorphogenesis is intriguing and not completely established. The JNK postsynaptic function is well recognized. In fact, JNK is rapidly activated after glutamate receptors stimulation [59], and in addition plays a key role in NMDA-induced neuronal-death (excitotoxicity; see [10, 60, 61]). Despite the great amount of data supporting JNK function after NMDA activation, nothing is known by now about JNK involvement in presynaptic NMDARs (pre-NMDARs) downstream pathway. The pre-NMDARs are present at the presynaptic site in the more plastic brain regions in adult, such as cortex and hippocampus [62], where they control neurotransmitter release [63, 64]. The activation of JNK signalling after NMDA stimulation at the presynaptic site is not proven yet, and only few studies have proposed a presynaptic role for JNK [65]. Many findings in literature show that synaptosomes preparation is a valid tool to value presynaptic compartment and related receptors, as previously described. By studying pre-NMDARs stimulation in synaptosomes, we showed for the first time JNK activation in the presynaptic site. Thanks to both microscopic and biochemical approaches, in fact, we detected JNK and its activated form, p-JNK, in cortical presynaptic terminals. Notably its levels in presynaptic site seem comparable to those of postsynaptic site, previously characterized ([17], see Figures 1 and 2). We proved and examined JNK activation after pre-NMDA receptors stimulation directly at pre-synaptic site (synaptosomes) in both young and adult cortex preparations. Interestingly, JNK activation rates were comparable for young and adult preparations, indicating that pre-NMDARs stimulation induced JNK activation in the presynaptic site at both ages. In addition, this process is conserved after neurodevelopment although pre-NMDARs expression is widely decreased and persisted only in cortex and hippocampus in adult [62]. No alteration of presynaptic proteins levels was observed in synaptosomes, suggesting that JNK is not involved in protein degradation, as what occurs in the postsynaptic compartment [17, 18, 30].

Literature evidences suggest that presynaptic kinases regulate neurotransmitter release, mainly affecting three steps: stored vesicle mobilization [66], axonal ending depolarization due to calcium influx [67], and presynaptic vesicles fusion [68]. In this context the role of JNK in regulating presynaptic tasks is poorly understood: there are only few data reporting vesicle mobilization as an important step in spontaneous release [69], while JNK inhibition had already been proven not to change calcium intracellular levels [25] suggesting that it is unlikely that the kinase could modulate calcium channels or NMDA receptors permeability. Thus thanks to our modelling design finding, we examined whether JNK acts as a physiological effector of SNARE complex formation regulating vesicle release. The biochemical data confirmed the prediction of the computational analysis, suggesting the interaction of JNK with Syntaxin-1, Syntaxin-2, and Snap25 as well as with the SNARE complex formation. We have demonstrated that Syntaxin-1 and Syntaxin-2 share around 64% of sequence identity and there is only one JBD motif fully conserved in both isoforms, while we found only one complete JBD motif into the Snap25 sequence. Once we got these data from the sequence alignment, we were able to map the JBD on the Syntaxin-1/Syntaxin-2 and Snap25 three-dimensional (3D) structures in order to perform the docking simulation. The results of the docking simulations can show the possible conformations of the Syntaxin-1/Syntaxin-2-JNK and Snap25-JNK complexes. However, the electrophysiological and biochemical experiments proved the rationale of the docking hypothesis.

To get more insight into JNK presynaptic function, we examined the effects of the specific JNK inhibitor D-JNKI1 on mEPSCs recorded from cortical slices. By preventing JNK action, the frequency, but not the amplitude, of mEPSCs was strongly reduced (50%) thus confirming a modulation of presynaptic release probability by JNK. These results combined with the biochemical findings suggest that the interaction of JNK with Syntaxin-1 and Syntaxin-2 as well as Snap25 at the presynaptic level could represent an important mechanism for regulating vesicle release.

Whether the JNK binding to Snap25 is direct or mediated by Syntaxin is difficult to establish since there are many interactions among the T-SNARE proteins that form the complex, including Syntaxin and Snap25 which, in resting conditions, are partially assembled in a primed structure [70–72]. For this reason, we evaluated NMDA-mediated SNARE complex assembly in presence of specific D-JNKI1 inhibitor. As expected, JNK inhibition reduces SNAREs complexation, indicating JNK as a modulator of vesicle docking-priming, via Syntaxin/Snap25 direct interactions and probably phosphorylation. Such effect may result in a fine regulation of neurotransmitter release by JNK signalling pathway.

We then tried to clarify which JNK isoforms are more prone to interact with Syntaxin-1/ Syntaxin-2 and Snap25. The brain specific JNK3 isoform, majorly related to NMDARs activation [7, 50], is the most intriguing form, but unfortunately the antibodies are not able to discriminate well among JNK isoforms. Immunoprecipitation assay suggests a functional overlapping between the two isoforms, JNK2 and JNK3. Further studies are needed to clarify this issue. These findings all together unravel a new and important role of JNK in the presynaptic site.

## 5. Conclusions

The JNK stress-signalling pathway is indicated as a key player in developmental and neurodegenerative pathologies. Its implication in excitotoxicity and synaptic dysfunction/dysmorphogenesis makes its importance clear. JNK represents a therapeutic target against neurodegenerative processes, but much information is missing to manipulate its action in order to reduce unwanted adverse effects. In this work we demonstrated for the first time that JNK leads to neurotransmitter release facilitation, due to a direct interaction with SNARE proteins. Thus an important new role of JNK in controlling synaptic plasticity emerges from this study, adding another part of the story to better clarify JNK function in synapse modulation.

## References

[1] Davis R. J. (2000). Signal transduction by the JNK group of MAP kinases. *Cell*.

[2] Chang L., Karin M. (2001). Mammalian MAP kinase signalling cascades. *Nature*.

[3] Angel P., Karin M. (1991). The role of Jun, Fos and the AP-1 complex in cell-proliferation and transformation. *BBA—Reviews on Cancer*.

[4] Kawauchi T., Chihama K., Nabeshima Y.-I., Hoshino M. (2003). The in vivo roles of STEF/Tiam1, Rac1 and JNK in cortical neuronal migration. *EMBO Journal*.

[5] Bogoyevitch M. A., Kobe B. (2006). Uses for JNK: the many and varied substrates of the c-Jun N-terminal kinases. *Microbiology and Molecular Biology Reviews*.

[6] Björkblom B., Östman N., Hongisto V. (2005). Constitutively active cytoplasmic c-Jun N-terminal kinase 1 is a dominant regulator of dendritic architecture: role of microtubule-associated protein 2 as an effector. *Journal of Neuroscience*.

[7] Kuan C.-Y., Whitmarsh A. J., Yang D. D. (2003). A critical role of neural-specific JNK3 for ischemic apoptosis. *Proceedings of the National Academy of Sciences of the United States of America*.

[8] Tararuk T., Östman N., Li W. (2006). JNK1 phosphorylation of SCG10 determines microtubule dynamics and axodendritic length. *Journal of Cell Biology*.

[9] Borsello T., Croquelois K., Hornung J.-P., Clarke P. G. H. (2003). N-methyl-D-aspartate-triggered neuronal death in organotypic hippocampal cultures is endocytic, autophagic and mediated by the c-Jun N-terminal kinase pathway. *European Journal of Neuroscience*.

[10] Borsellol T., Clarkel P. G. H., Hirt L. (2003). A peptide inhibitor of c-Jun N-terminal kinase protects against excitotoxicity and cerebral ischemia. *Nature Medicine*.

[11] Repici M., Centeno C., Tomasi S. (2007). Time-course of c-Jun N-terminal kinase activation after cerebral ischemia and effect of D-JNKI1 on c-Jun and caspase-3 activation. *Neuroscience*.

[12] Tran H. T., Sanchez L., Brody D. L. (2012). Inhibition of JNK by a peptide inhibitor reduces traumatic brain injury-induced tauopathy in transgenic mice. *Journal of Neuropathology and Experimental Neurology*.

[13] Yoon S. O., Park D. J., Ryu J. C. (2012). JNK3 perpetuates metabolic stress induced by A*β* peptides. *Neuron*.

[14] Savage M. J., Lin Y.-G., Ciallella J. R., Flood D. G., Scott R. W. (2002). Activation of c-Jun N-terminal kinase and p38 in an Alzheimer's disease model is associated with amyloid deposition. *Journal of Neuroscience*.

[15] Reynolds C. H., Betts J. C., Blackstock W. P., Nebreda A. R., Anderton B. H. (2000). Phosphorylation sites on tau identified by nanoelectrospray mass spectrometry: differences in vitro between the mitogen-activated protein kinases ERK2, c-Jun N-terminal kinase and P38, and glycogen synthase kinase-3*β*. *Journal of Neurochemistry*.

[16] Braithwaite S. P., Schmid R. S., He D. N. (2010). Inhibition of c-Jun kinase provides neuroprotection in a model of Alzheimer's disease. *Neurobiology of Disease*.

[17] Sclip A., Arnaboldi A., Colombo I. (2013). Soluble A*β* oligomer-induced synaptopathy: c-Jun N-terminal kinase's role. *Journal of Molecular Cell Biology*.

[18] Sclip A., Antoniou X., Colombo A. (2011). c-Jun N-terminal kinase regulates soluble A*β* oligomers and cognitive impairment in AD mouse model. *Journal of Biological Chemistry*.

[19] Liu Y. F. (1998). Expression of polyglutamine-expanded Huntingtin activates the SEK1-JNK pathway and induces apoptosis in a hippocampal neuronal cell line. *Journal of Biological Chemistry*.

[20] Ferrer I., Carmona M., Puig B., Domínguez I., Viñals F. (2002). Active, phosphorylation-dependent MAP kinases, MAPK/ERK, SAPK/JNK and p38, and specific transcription factor substrates are differentially expressed following systemic administration of kainic acid to the adult rat. *Acta Neuropathologica*.

[21] Ferrer I., Blanco R., Carmona M., Puig B. (2001). Phosphorylated mitogen-activated protein kinase (MAPK/ERK-P), protein kinase of 38kDa (p38-P), stress-activated protein kinase (SAPK/JNK-P), and calcium/calmodulin-dependent kinase II (CaM kinase II) are differentially expressed in tau deposits in neurons and glial cells in tauopathies. *Journal of Neural Transmission*.

[22] Coffey E. T. (2014). Nuclear and cytosolic JNK signalling in neurons. *Nature Reviews Neuroscience*.

[23] Coffey E. T., Hongisto V., Dickens M., Davis R. J., Courtney M. J. (2000). Dual roles for c-Jun N-terminal kinase in developmental and stress responses in cerebellar granule neurons. *Journal of Neuroscience*.

[24] Björkblom B., Vainio J. C., Hongisto V., Herdegen T., Courtney M. J., Coffey E. T. (2008). All JNKs can kill, but nuclear localization is critical for neuronal death. *Journal of Biological Chemistry*.

[25] Centeno C., Repici M., Chatton J.-Y. (2007). Role of the JNK pathway in NMDA-mediated excitotoxicity of cortical neurons. *Cell Death and Differentiation*.

[26] Westerlund N., Zdrojewska J., Padzik A. (2011). Phosphorylation of SCG10/stathmin-2 determines multipolar stage exit and neuronal migration rate. *Nature Neuroscience*.

[27] Kim M. J., Futai K., Jo J., Hayashi Y., Cho K., Sheng M. (2007). Synaptic accumulation of PSD-95 and synaptic function regulated by phosphorylation of serine-295 of PSD-95. *Neuron*.

[28] Yang H., Courtney M. J., Martinsson P., Manahan-Vaughan D. (2011). Hippocampal long-term depression is enhanced, depotentiation is inhibited and long-term potentiation is unaffected by the application of a selective c-Jun N-terminal kinase inhibitor to freely behaving rats. *European Journal of Neuroscience*.

[29] Farías G. G., Alfaro I. E., Cerpa W. (2009). Wnt-5a/JNK signaling promotes the clustering of PSD-95 in hippocampal neurons. *Journal of Biological Chemistry*.

[30] Thomas G. M., Lin D.-T., Nuriya M., Huganir R. L. (2008). Rapid and bi-directional regulation of AMPA receptor phosphorylation and trafficking by JNK. *EMBO Journal*.

[31] Sclip A., Tozzi A., Abaza A. (2014). c-Jun N-terminal kinase has a key role in Alzheimer disease synaptic dysfunction *in vivo*. *Cell Death & Disease*.

[32] Mukherjee P. K., DeCoster M. A., Campbell F. Z., Davis R. J., Bazan N. G. (1999). Glutamate receptor signaling interplay modulates stress-sensitive mitogen-activated protein kinases and neuronal cell death. *Journal of Biological Chemistry*.

[33] Corlew R., Brasier D. J., Feldman D. E., Philpot B. D. (2008). Presynaptic NMDA receptors: newly appreciated roles in cortical synaptic function and plasticity. *Neuroscientist*.

[34] Rodríguez-Moreno A., Banerjee A., Paulsen O. (2010). Presynaptic NMDA receptors and spike timing-dependent depression at cortical synapses. *Frontiers in Synaptic Neuroscience*.

[35] Larsen R. S., Smith I. T., Miriyala J. (2014). Synapse-specific control of experience-dependent plasticity by presynaptic NMDA receptors. *Neuron*.

[36] Banerjee A., Larsen R. S., Philpot B. D., Paulsen O. (2016). Roles of Presynaptic NMDA Receptors in Neurotransmission and Plasticity. *Trends in Neurosciences*.

[37] Brasier D. J., Feldman D. E. (2008). Synapse-specific expression of functional presynaptic NMDA receptors in rat somatosensory cortex. *Journal of Neuroscience*.

[38] Fedder K., Sabo S. (2015). On the role of glutamate in presynaptic development: possible contributions of presynaptic NMDA receptors. *Biomolecules*.

[39] Kunz P. A., Roberts A. C., Philpot B. D. (2013). Presynaptic NMDA receptor mechanisms for enhancing spontaneous neurotransmitter release. *Journal of Neuroscience*.

[40] Cull-Candy S., Brickley S., Farrant M. (2001). NMDA receptor subunits: diversity, development and disease. *Current Opinion in Neurobiology*.

[41] Pérez-Otaño I., Ehlers M. D. (2004). Learning from NMDA receptor trafficking: clues to the development and maturation of glutamatergic synapses. *NeuroSignals*.

[42] Lau C. G., Zukin R. S. (2007). NMDA receptor trafficking in synaptic plasticity and neuropsychiatric disorders. *Nature Reviews Neuroscience*.

[43] Corlew R., Wang Y., Ghermazien H., Erisir A., Philpot B. D. (2007). Developmental switch in the contribution of presynaptic and postsynaptic NMDA receptors to long-term depression. *Journal of Neuroscience*.

[44] Yang J., Woodhall G. L., Jones R. S. G. (2006). Tonic facilitation of glutamate release by presynaptic NR2B-containing NMDA receptors is increased in the entorhinal cortex of chronically epileptic rats. *Journal of Neuroscience*.

[45] Frasca A., Aalbers M., Frigerio F. (2011). Misplaced NMDA receptors in epileptogenesis contribute to excitotoxicity. *Neurobiology of Disease*.

[46] Evans G. J. O. (2015). Subcellular fractionation of the brain: preparation of synaptosomes and synaptic vesicles. *Cold Spring Harbor Protocols*.

[47] Bonny C., Oberson A., Steinmann M., Schorderet D. F., Nicod P., Waeber G. (2000). IB1 reduces cytokine-induced apoptosis of insulin-secreting cells. *Journal of Biological Chemistry*.

[48] Burkhardt P., Hattendorf D. A., Weis W. I., Fasshauer D. (2008). Munc18a controls SNARE assembly through its interaction with the syntaxin N-peptide. *EMBO Journal*.

[49] De Vries S. J., Van Dijk M., Bonvin A. M. J. J. (2010). The HADDOCK web server for data-driven biomolecular docking. *Nature Protocols*.

[50] Yang D. D., Kuan C.-Y., Whitmarsh A. J. (1997). Absence of excitotoxicity-induced apoptosis in the hippocampus of mice lacking the Jnk3 gene. *Nature*.

[51] Falace A., Buhler E., Fadda M. (2014). TBC1D24 regulates neuronal migration and maturation through modulation of the ARF6-dependent pathway. *Proceedings of the National Academy of Sciences of the United States of America*.

[52] Liu Z.-W., Faraguna U., Cirelli C., Tononi G., Gao X.-B. (2010). Direct evidence for wake-related increases and sleep-related decreases in synaptic strength in rodent cortex. *Journal of Neuroscience*.

[53] Burré J., Sharma M., Tsetsenis T., Buchman V., Etherton M. R., Südhof T. C. (2010). *α*-synuclein promotes SNARE-complex assembly in vivo and in vitro. *Science*.

[54] Seo J., Hong J., Lee S. J., Choi S.-Y. (2012). c-Jun N-terminal phosphorylation is essential for hippocampal synaptic plasticity. *Neuroscience Letters*.

[55] Mao L.-M., Wang J. Q. (2015). Synaptically localized mitogen-activated protein kinases: local substrates and regulation. *Molecular Neurobiology*.

[56] Kim H.-S., Park C. H., Cha S. H. (2000). Carboxyl-terminal fragment of Alzheimer's APP destabilizes calcium homeostasis and renders neuronal cells vulnerable to excitotoxicity. *FASEB Journal*.

[57] Choo A. M., Geddes-Klein D. M., Hockenberry A. (2012). NR2A and NR2B subunits differentially mediate MAP kinase signaling and mitochondrial morphology following excitotoxic insult. *Neurochemistry International*.

[58] Paoletti P., Bellone C., Zhou Q. (2013). NMDA receptor subunit diversity: impact on receptor properties, synaptic plasticity and disease. *Nature Reviews Neuroscience*.

[59] Tian H., Zhang Q.-G., Zhu G.-X., Pei D.-S., Guan Q.-H., Zhang G.-Y. (2005). Activation of c-Jun NH_2_-terminal kinase 3 is mediated by the GluR6*∙*PSD-95*∙*MLK3 signaling module following cerebral ischemia in rat hippocampus. *Brain Research*.

[60] Shi Z.-Q., Sunico C. R., McKercher S. R. (2013). S-nitrosylated SHP-2 contributes to NMDA receptor-mediated excitotoxicity in acute ischemic stroke. *Proceedings of the National Academy of Sciences of the United States of America*.

[61] Lipton S. A. (2006). Paradigm shift in neuroprotection by NMDA receptor blockade: memantine and beyond. *Nature Reviews Drug Discovery*.

[62] Bouvier G., Bidoret C., Casado M., Paoletti P. (2015). Presynaptic NMDA receptors: roles and rules. *Neuroscience*.

[63] Pittaluga A., Feligioni M., Longordo F., Arvigo M., Raiteri M. (2005). Somatostatin-induced activation and up-regulation of N-methyl-D-aspartate receptor function: mediation through calmodulin-dependent protein kinase II, phospholipase C, protein kinase C, and tyrosine kinase in hippocampal noradrenergic nerve endings. *Journal of Pharmacology and Experimental Therapeutics*.

[64] Musante V., Summa M., Cunha R. A., Raiteri M., Pittaluga A. (2011). Pre-synaptic glycine GlyT1 transporter—NMDA receptor interaction: relevance to NMDA autoreceptor activation in the presence of Mg^2+^ ions. *Journal of Neurochemistry*.

[65] Nisticò R., Florenzano F., Mango D. (2015). Presynaptic c-Jun N-terminal Kinase 2 regulates NMDA receptor-dependent glutamate release. *Scientific Reports*.

[66] Giachello C. N. G., Fiumara F., Giacomini C. (2010). MAPK/Erk-dependent phosphorylation of synapsin mediates formation of functional synapses and short-term homosynaptic plasticity. *Journal of Cell Science*.

[67] Martin S. W., Butcher A. J., Berrow N. S. (2006). Phosphorylation sites on calcium channel *α*1 and *β* subunits regulate ERK-dependent modulation of neuronal N-type calcium channels. *Cell Calcium*.

[68] Liu T., Tucker W. C., Bhalla A., Chapman E. R., Weisshaar J. C. (2005). SNARE-driven, 25-millisecond vesicle fusion in vitro. *Biophysical Journal*.

[69] Lin J.-W., Sugimori M., Llinás R. R., McGuinness T. L., Greengard P. (1990). Effects of synapsin I and calcium/calmodulin-dependent protein kinase II on spontaneous neurotransmitter release in the squid giant synapse. *Proceedings of the National Academy of Sciences of the United States of America*.

[70] Ohara-Imaizumi M., Nishiwaki C., Nakamichi Y., Kikuta T., Nagai S., Nagamatsu S. (2004). Correlation of syntaxin-1 and SNAP-25 clusters with docking and fusion of insulin granules analysed by total internal reflection fluorescence microscopy. *Diabetologia*.

[71] Xiao J., Xia Z., Pradhan A., Zhou Q., Liu Y. (2004). An immunohistochemical method that distinguishes free from complexed SNAP-25. *Journal of Neuroscience Research*.

[72] Vogel K., Cabaniols J.-P., Roche P. A. (2000). Targeting of SNAP-25 to membranes is mediated by its association with the target SNARE syntaxin. *Journal of Biological Chemistry*.

